# Announcement: Seventh International Conference on Bioinorganic Chemistry ICBIC7

**DOI:** 10.1155/MBD.1994.347

**Published:** 1994


ICBIC7

LÜBECK'95

### Seventh International Conference on Bioinorganic Chemistry to be held in the old City of Lübeck, Germany

**3 - 8 September 1995**

### Introduction

The Organizing Committee is pleased to invite all interested scientists to attend ICBIC 7. This Conference continues the biennial tradition of conferences held in Florence, Italy (1983), Algarve, Portugal (1985), Noordwijkerhout, The Netherlands (1987), Boston, USA (1989), Oxford. UK (1991) and San Diego, USA (1993).

An informal evening reception, on Sunday 3, September, will precede the scientific sessions. Plenary lectures are followed by parallel sessions and poster presentations on a variety of contemporary research topics. For Wednesday afternoon optional excursions are planned. The conference will end with a conference dinner Friday evening, 8 September.

The program will be organized around eight plenary lectures, which will introduce topics to be expanded by invited speakers in two parallel sessions and additional microsymposia, and by two poster sessions.

**Tentative topics include:**
- **Redox reactions**
- **Hydrolytic and group transfer**
- **Energy transfer, bioenergetics**
- **Transport, storage, and assembly of metals**
- **Metals and nucleic acids**
- **Gene regulation**
- **Metals in medicine**
- **Environmental chemistry**
- **Spectroscopy and specific applications** and more

### Plenary Lectures

- Stephen Lippard (Cambridge):
Synthetic models for and mechanistic studies of methane monooxygenase
- Ken Raymond (Berkeley)
The coordination chemistry of biological iron transport: Iron and disease

• Dieter Sellmann (Erlangen)
Modelling the reactivity of metal-sulfur oxidoreductases
• Britt-Marie Sjöberg (Stockholm):
Ribonucleotide reductase - an ancient enzyme with radical mechanism
• Rolf Thauer (Marburg)
Metalloenzymes involved in methanogenesis
• Andy Thomson (Norwich)
Magneto-optics and metalloproteins
• Anthony Wedd (Melbourne)
Oxo-molybdenum enzymes
• Raymond Weiss (Strasbourg)
Advances in modelling the high-valent iron intermediates of heme proteins

**Poster Sessions**

The Conference will continue the tradition of poster sessions to disseminate original and significant research in progress. There will be ample time for poster presentation because we plan to organize each poster session as two-day exhibition.

The Conference solicits contributions. Because of space limitation please plan to limit yourself to a single poster.

Call for posters: Formats required to prepare posters and to publish abstracts will be sent with the second announcement. Abstracts will be accepted for publication in Journal of Inorganic Biochemistry only after receipt of conference fee.

**Social Events**

• Get-together party on Sunday evening, 3 September.
• Organ concert at Marienkirche, the famous place of Bach's and Buxtehude's church music, on Tuesday evening, 5 September. Optional excursions to the old City of Lübeck, to Mecklenburg, to Schleswig-Holstein, to Hamburg etc., on Wednesday afternoon, 6 September.
• Official Conference Dinner on Friday evening, 8 September.

We are planning a panorama of daytime events for accompanying persons who are not engaged in the scientific program. Details will be included in the second announcement.

**Location**

The conference will take place at the new music and congress hall of Lübeck, next to thehistorical Holstentor (which we chose as conference logo for ICBIC 7), and in walking distance to the old city and to several hotels.

Lübeck is located 60 km east of Hamburg at the shore of the Baltic Sea. The most convenient airport is Hamburg. A freeway and railway system links Lübeck by land. Additionally, there are numerous ferry links from Travemünde (the port of Lübeck) to Scandinavia, Poland and Russia.

The history of Lübeck goes back to around the year 1000 A.D. The great medieval churches - St. Mary's, St. Peter's, St. Jacob's, St. Ägidien's and the Cathedral - were built in the 12th and 13th centuries together with many other monuments which impress our visitors, as well as ourselves still every day. The old City - former capital of the Hanseatic League - is an architectural museum of the middle ages, and it has just been elected "UNESCO World Heritage Site". But you will hopefully notice that this museum is full of life.

**Fees**

A preliminary estimate of the registration fees (conference abstracts and printed material, refreshments, Sunday evening reception, Tuesday evening organ concert, Friday evening conference dinner):

| | Before | After |
|---|---|---|
| | June 1, 1995 | |
| Participant | 700.— DM | 900.— DM |
| Graduate student*, Postdoc* | 400.— DM | 600.— DM |
| Accompanying Person | 200.— DM | 300.— DM |

* with documentation

A registration form will be included in the second announcement.

**Accomodation**

Accomodation will be provided in hotels close to conference site. The conference office will offer three categories.
Arrangements may be made to arrive before and/or to stay after the meeting on a daily rate basis.

Several fine hotels are located near the conference site. Blocks of rooms have been set aside. Accomodation form will be included in the second announcement.

**Committees**

*Conference Chair*
Alfred X. Trautwein (Lübeck) and Karl Wieghardt (Bochum)

*National Organizing Committee*

Wolfgang Buckel (Marburg), Bärbel Friedrich (Berlin), Bernt Krebs (Münster), Peter Kroneck (Konstanz), Bernhard Lippert (Dortmund), Heinrich Vahrenkamp (Freiburg), Günter Winkelmann (Tübingen), Michael Zeppezauer (Saarbrücken)

*Local Organizing Committee*
Stefan Anemüller, Ute Baumhöver, Eckhard Bill, Veronika Damaschke, Karl-Heinz Finder, Andrzey Sawaryn, Axel Schiller, Heiner Winkler (Lübeck), Phalguni Chaudhuri (Bochum), Hans-Jörg Krüger (Hamburg)

**Funds for travelling**

Probably a limited amount of money will be available to cover at least partly registration fees of EastEuropean collegues who attend ICBIC 7. Informal applications should be sent to Alfred X. Trautwein.

Those who want to receive the second announcement or any additional information should phone, fax or write to

**Alfred X. Trautwein**

**ICBIC 7**

**Institut für Physik, Medizinische Universität**

**RatzeburgerAllee 160**

**D-23538 Lübeck, Germany**

**Tel.: ++ 49 451 500 4200**
**Fax: ++49 451 500 4214**
**e-mail: icbic7@miraculix.physik.mu-luebeck.de**

**or to Karl Wieghardt**
**Tel.: ++49 234 700 4153**
**Fax: ++49 234 700 4109**

